# Effects of Incubation Light on Behaviour, Growth Performance, Blood Parameters, and Digestive Enzymes in Post-Hatch Layer Chicks

**DOI:** 10.3390/ani14152197

**Published:** 2024-07-28

**Authors:** Peng Yin, Siqi Wei, Qin Tong, Baoming Li, Weichao Zheng, Xiaoliu Xue, Chenxin Shi

**Affiliations:** 1 Department of Agricultural Structure and Environmental Engineering, College of Water Resources and Civil Engineering, China Agricultural University, Beijing 100083, China; yinpeng@cau.edu.cn (P.Y.); weisiqi477@cau.edu.cn (S.W.); tongqin@cau.edu.cn (Q.T.); weichaozheng@cau.edu.cn (W.Z.); xuexiaoliu@cau.edu.cn (X.X.); shichenxin@cau.edu.cn (C.S.); 2 Key Laboratory of Agricultural Engineering in Structure and Environment, Ministry of Agriculture and Rural Affairs, Beijing 100083, China; 3 Beijing Engineering Research Center on Animal Healthy Environment, Beijing 100083, China

**Keywords:** chick, incubation light, behaviour, growth performance, hormones

## Abstract

**Simple Summary:**

In commercial hatcheries, chicken embryos are incubated in complete darkness. However, in a natural environment, chicken embryos receive light stimulation during the incubation process as hens need to leave the nest to feed or turn the eggs. Therefore, providing light during incubation is closer to the natural growth environment of chicken embryos, which may have a positive effect on their growth and development. This study investigated the effects of providing different wavelengths of light during incubation on the behaviour, growth, immunity, and stress responses of post-hatch chicks. The results indicated that providing white light during incubation may enhance the growth performance of post-hatch chicks and potentially improve their ability to cope with environmental changes. This study aims to determine the effects of light exposure during incubation on the growth and development of post-hatch chicks and to provide a basis for the development of effective lighting strategies during the incubation period.

**Abstract:**

Manipulation of light during incubation may have an effect on post-hatch chicks through the role of prenatal stage. The effects of providing different wavelengths of light (white, blue, and green lights, dark as control) during incubation on the growth performance, organ development, immune response, stress related hormones, digestive enzymes and behaviour of post-hatch chicks were investigated for 1–42 days. A total of 60 chicks per light treatment in three batches were used in this study. The results showed that the percentage of chicks accessing to feed and water resources appeared not to be affected by incubation light. Chicks hatched under white light were found to have a growth advantage (*p* < 0.05). The weight of organs (except thymus), IgA, IgY, IgM and heterophil to lymphocyte (H/L) ratio for post-hatch chicks were not affected by incubation light (*p* > 0.05). Thymus weight was reduced in chicks incubated under blue light compared to dark incubation (*p* < 0.05). The jejunum amylase and ileum lipase activities were significantly affected by the light treatments (*p* < 0.01). All light incubation chicks had stable plasma corticosterone levels and may have better ability to cope with environmental changes. Hence, white light photoperiod incubation may have potential to improve post-hatch chicks’ growth performance and environmental adaptability.

## 1. Introduction

Lighting is one of the most important environmental factors in poultry production. Light stimulation significantly impacts poultry growth, reproductive performance, and overall health [1,2,3]. In the natural environment, because hens need to leave the nest to feed [4] or turn the eggs [5], chicken embryos receive light stimulation during the incubation process. Therefore, providing light during incubation is closer to the natural growth environment of chicken embryos compared to the current practice of incubating in darkness.

Existing research has indicated that retinal photoreceptors in chicken embryos begin responding to light/dark cycles by producing melatonin from ED16 (embryonic day) [6], and the visual system starts functioning by ED18, maturing further post-hatching [7]. Recent evidence has suggested that light may influence embryonic neurodevelopment from the outset of the incubation period, with responses to light occurring as early as ED3 through gene activation [8,9]. Early light stimulation during incubation (ED1–21) can further promote embryonic length development and shorten the hatch window compared to mid-to-late incubation light stimulation (ED7–21, ED14–21) [10], and chicken embryos can selectively respond to different wavelengths of light during incubation [11].

Light exposure during the incubation process has been demonstrated to alter embryonic physiological functions and lead to changes in behaviour, growth, and stress responses in post-hatch chicks [12,13,14]. As previously reported, green light during incubation suppressed the expression of green and red opsin genes in the final three days before hatching, with this suppressive effect continuing until the ninth day post-hatching [15]. Incubation light also affects the lateralisation of the visual system in chicken embryos, subsequently influencing the chicks’ perception of and interaction with their environment post-hatching [16]. Previous research has shown that chicks hatched under white light exhibit stronger visual learning abilities [17] and positive effects on the development of normal pecking behaviour and the ability to distinguish between edible and non-edible objects [18], although it has a negative effect on the development of feather pecking behaviour [14]. Light exposure during incubation can also influence cellular proliferation in embryos and promote the growth and development of chicks post-hatching [19]. Green light (560 nm) during incubation can increase feed intake and promote body weight gain in chicks from 0 to 6 days post-hatching, particularly enhancing breast muscle development at 3 and 6 days of age [13]. Incubation light can also alter melatonin rhythms and influence the stress response in chicks post-hatching [20]. Reports suggested that compared to dark incubation, white light during incubation results in lower increases in plasma corticosterone (CORT) levels within 8 hours post-hatching and a reduced stress response in chicks [21]. Archer [12] compared four stress indicators in chicks at 42 days post-hatching, finding that white or red light (630 nm) during incubation significantly reduced plasma CORT levels and increased serotonin levels compared to dark incubation and green light (520 nm). This suggests that incubation light may have a lasting effect on chicks. However, another study indicated that while there were differences in hormone levels in the plasma of embryos incubated under white light and darkness on the 19th day post-hatching, no differences were observed at the 5th week [22].

Currently, the effects of using different wavelengths of light during incubation on chick behaviour, growth, and stress responses, as well as the characteristics of these effects over time, remain unclear. This study aimed to investigate the impact of a 12L:12D lighting schedule with blue, green, and white light during incubation on behaviour of accessing to feed or water, body weight changes, organ development, plasma CORT, serum immunoglobulin, and digestive enzymes activity in chicks from 1 to 42 days old. The objective was to determine the impact of incubation lighting on post-hatch chick development and to provide a basis for the development of effective lighting strategies during the incubation period.

## 2. Materials and Methods

### 2.1. Animals and Management

The experiment was carried out in 3 batches. The incubation period was 21 d, of which 1 to 19 d were carried out in the setter (EIFXDZ-75600, Xingyi Electronic Equipment Co., Ltd., Qingdao, China) and 19 to 21 d (457–504 h) were in the hatcher (EICXDH-15120, Xingyi Electronic Equipment Co., Ltd., Qingdao, China). A total of 504 brown-shell eggs (Jinghong No. 1, Beijing Huadu Yukou Poultry Industry Co., Ltd., Beijing, China) were randomly divided into 4 groups for each batch and incubated under 4 different lighting treatments. Throughout the incubation period (ED0–21), a 12L:12D lighting schedule was used with monochromatic green light (525 nm/515–535 nm), monochromatic blue light (455 nm/447.5–462.5 nm), and white light (4000 K) as the light groups, and no light (darkness) as the control group. The light period consisted of 4 h of low intensity (200 ± 50 clux), followed by 1 h of continuous illumination at a high intensity (2000 ± 500 clux) and the remaining 7 h at low intensity (200 ± 50 clux). The detailed incubation procedure can be referred to in the study by Tang et al. [23]. At ED19, eggs were candled to remove non-fertile eggs and the remaining fertile eggs were transferred to the hatcher. At the end of the 504 h, the hatching period was terminated.

After hatching, 80 female chicks (20 chicks from each group per batch) were selected, tagged with animal foot rings, and transferred to the poultry laboratory at China Agricultural University. They were allocated into four groups of pens according to the different light treatments groups and reared until 42 days of age. The dimensions of the individual pen were 0.96 m L × 1.2 m W × 2.0 m H. The pens were separated by PVC boards. Each pen was equipped with a feeder and a bell drinker. The floor of each pen was lined with plastic mesh, and manure was collected using manure trays. Manure was removed daily. Each pen was illuminated by two 7 W white LED lights (LT-DB75P, Hangzhou LightTalk Biotechnology, Co., Ltd., Hangzhou, China) installed at the top. Lights were on at 60 lx from 0:00–23:00 at D1–D3 (the first three days after hatching) and at 30 lx from 1:00–23:00 at D4–D7. Then this was followed by an even reduction of two hours per week, i.e., adjusted from 2:00–22:00 at 20 lx in the second week (D8–D14) to 6:00–18:00 in the sixth week (D36–D42). A camera (Zhejiang Dahua Technology Co., Ltd., Hangzhou, China) was installed at the top of each pen to record the feeding and drinking behaviour of the chicks. The acquired video resolution was 1280 × 720 pixels, and the camera’s frame rate was 15 fps. The environmental conditions for chicks in the brooding period followed industrial recommendations, and both temperature and humidity were monitored and controlled at appropriate levels.

### 2.2. Behaviour of Chicks Accessing to Feed or Water

The collection of chick behaviour videos was conducted from 1:00 to 23:00 during the first week of each batch. To accurately detect the behaviour of chicks accessing to feed or water, a detection model was developed based on the YOLO v4 object detection algorithm. An initial dataset was created by randomly extracting 3600 frames from the chick behaviour videos. The chicks accessed to the feeder or drinker, as well as the feeder and drinker themselves, were annotated in the initial dataset using annotation tools. The dataset was then divided into training, validation, and test sets in a ratio of 8:1:1. Model training was performed for a total of 1000 epochs and the best model was selected based on the loss in the training and validation sets. The test set was identified using the best model, and the indicators of precision, recall, average precision (AP), and mean average precision (mAP) were selected to evaluate the performance of the model.

**Figure 1:**
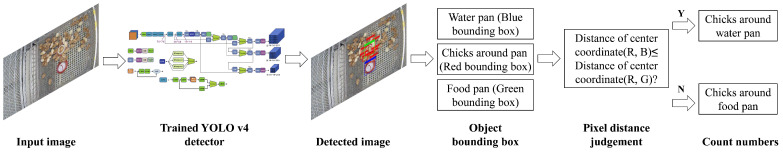
The workflow of the overall algorithms for automatically obtaining the number of chicks accessing to the feeder and drinker.

The workflow of identifying the chicks accessed to the feed and water is shown in Figure 1. First, an image was fed into the trained behaviour detector. Trained detector would output different colour detection boxes depending on the objects in the image. Red represented that the chicks was in close proximity to resources, blue represented the bell drinker, and green represented the feeder. Then, the centre distance between the red boxes and the green box or the blue box was calculated. If the distance between the centre of the red box and the green box was shorter than the distance between the centre of the red box and the blue box, the chick was identified as accessing to the feeder. Otherwise, the chick was identified as accessing to the drinker. Finally, the program was used to output the total number of chicks that accessed to feed or water in the image, respectively.

Videos of each group from three batches during 9:55–10:00 (M), 12:55–13:00 (N), and 15:55–16:00 (A) at D1, D4, and D7 were intercepted. Each five-minute video was converted into 300 frames at 1-s intervals. The chick recognition model was used to obtain the number of chicks accessing to the feeder and drinker in each image. Then the mean percentage of chicks accessing to feed or water out of the total chicks in the five minutes of different period was counted.

### 2.3. Growth Performance, Blood Hormones, and Digestive Enzyme Activity

All chicks of each group from three batches were weighed every day at the age of 1–7 days and every week at the age of 14 to 42 days. On D21 and D42, three chicks of each group were randomly selected and were weighed individually before being euthanized through cervical dislocation. Blood samples were collected from the neck vessels immediately after euthanasia. A drop of whole blood was smeared on a glass slide, which was stained using a hematology staining kit (Beijing Huaying Biotechnology Research Institute, Beijing, China), air dried and stored in a slide box. One hundred leukocytes per slide were counted twice and the heterophil to lymphocyte (H/L) ratio was calculated. Furthermore, about 3–5 mL of blood from samples was collected into heparin tubes and centrifuged at 3000 rpm for 10 min. The plasma was separated and stored at −20 °C until further analysis. IgY, IgA, and IgM were measured using the HY-50091 kit (Beijing Huaying Biotechnology Research Institute, Beijing, China). The plasma CORT was analysed using Cort HY-1006 kit (Beijing Huaying Biotechnology Research Institute, Beijing, China). After blood sampling, the glandular stomach (chyme removed), muscle stomach (chyme removed), pancreas, spleen, thymus, bursa, liver, and heart were excised and weighed (g). The contents of jejunum and ileum from samples of last batch were intercepted and stored at −30 °C and homogenized in a solution of 100 mM mannitol and 2 mM HEPES/KOH (pH 6.5), centrifuged at 2200× *g* for 10 min. To measure the activity of amylase (EC 3.2.1.1) in jejunum and the activity of lipase (EC 3.1.1.3) in ileum, the aliquots of the supernatant were analysed using diagnostic kits (HY-N0045 and HY-60115 kits, Beijing Huaying Biotechnology Research Institute, Beijing, China) according to the manufacturer’s protocol. The activity of amylase was expressed as U/g protein, while lipase was U/mg protein.

### 2.4. Statistical Analysis

Data were analysed using IBM SPSS Statistics 25 (IBM Corporation, Armonk, USA) and presented as the mean ± SEM. All data were tested using the Shapiro–Wilk test (normal distribution test) and F-test (homogeneity of variances test). If the data did not conform to a normal distribution, the original data were processed using square root inverse sine transform.

A linear mixed model was used to analyse the effect of incubation light treatments on body weight.

Y_ijk_ = μ+ L_i_ + A_j_ + B_k_+ L(A)_ij_ + L(B)_ik_ + A(B)_jk_ + e_ijk_ (1)

where Y_ijk_ is the body weight; µ is the overall mean; L_i_ is the fixed effect of incubation light (i = control, blue, green, white); A_j_ is the fixed effect of age (j = D1-D7, D14/21/28/35/42); B_k_ is the random effect of batch (k = 1–3); L(A)_ij_, L(B)_ik_, and A(B)_jk_ are the interaction effects; and e_ijk_ is the error effect.

A second linear mixed model was used to analyse the effect of incubation light on organ weight, lgA, lgG, lgM, H/L ratio, jejunum amylase activity, ileum lipase activity, and plasma CORT levels.

Y_ij_ = μ+ L_i_ + A_j_ + L(A)_ij_ + e_ijk_ (2)

where Y_ij_ is organ weight, lgA, lgG, lgM, H/L ratio, jejunum amylase activity, ileum lipase activity, and plasma CORT levels; µ is the overall mean; L_i_ is the fixed effect of light (i = control, blue, green, white); A_j_ is the fixed effect of age (j= D21, D42); L(A)_ij_ is the interaction effect; and e_ijk_ is the error effect.

A third linear mixed model was used to analyse the effect of incubation light on processed percentage of chicks accessing to the feed or water.

Y_ijkl_ = μ + L_i_ + A_j_ + B_k_+ S_l_+ L(A)_ij_ + L(B)_ik_ + L(S)_il_ + A(B)_jk_ + A(S)_jl_ + B(S)_kl_ + e_ijkl_ (3)

where Y_ijkl_ is the processed percentage of chicks accessing to the feed or water; µ is the overall mean; L_i_ is the fixed effect of light (i = control, blue, green, white); A_j_ is the fixed effect of age (j = D1, D4, D7); B_k_ is random effect of batch (k = 1–3); S_l_ is the fixed effect of sampling time (l = M, N, A); L(A)_ij_, L(B)_ik_, L(S)_il_, A(B)_jk_, A(S)_jl_, and B(S)_kl_ are the interaction effects; and e_ijkl_ is the error effect.

When the effect was statistically significant (p < 0.05), the means were further analyzed using LSD test or nonparametric statistics.

## 3. Results

### 3.1. Proportion of Chicks Accessing to Feed or Water

There was no overfitting during model training. The model trained to the 997th epoch was selected for subsequent trials with a model loss of 0.027. The performance of the model on the test dataset is shown in Table 1. The mAP of trained model was 97.64%. The model demonstrated good performance in automatically detecting chicks, feeder, and drinker. Based on this model and the workflow for chicks accessing to the feeder and drinker (Figure 1), the number of chicks in the video samples was automatically identified and the output results were saved.

**Figure 2:**
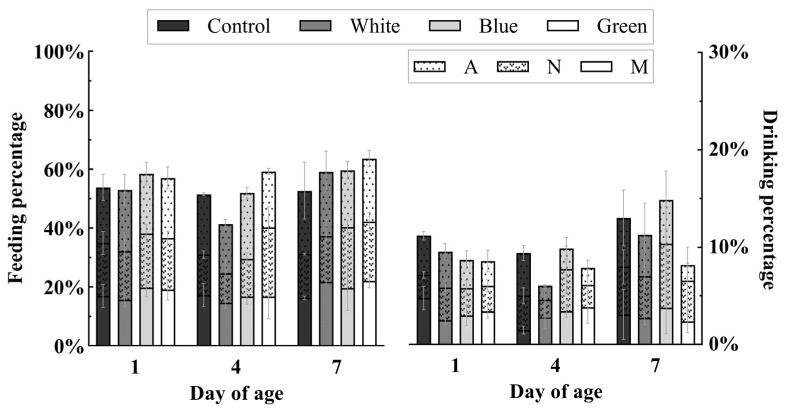
Percentage of chicks hatched under different incubation light treatments accessed to feed and water at different times on D1, D4, and D7. M, N, and A each represent different sampling times, specifically 9:55–10:00 (M), 12:55–13:00 (N), and 15:55–16:00 (A).

Data analysis was conducted on the output results of the model. The results indicated that batch, age, sampling time and light treatments had no significant effect on the proportion of chicks accessing to the feeder or drinker (*p* > 0.05). The proportion of chicks of different ages accessing to the feeder or drinker at different times of the day is shown in Figure 2. There was no difference in percentage of chicks accessing to feed or water between light treatments on D1, D4 and D7. The mean percentages of chicks accessing feed at 9:55–10:00 (M), 12:55–13:00 (N), and 15:55–16:00 (A) were 17.93% ± 1.00%, 16.66% ± 1.18%, and 20.22% ± 0.93%, respectively, while percentages of chicks accessing water were 3.09% ± 0.32%, 3.41% ± 0.42%, and 3.06% ± 0.36%, respectively.

### 3.2. Growth Performance of Chicks

**Figure 3:**
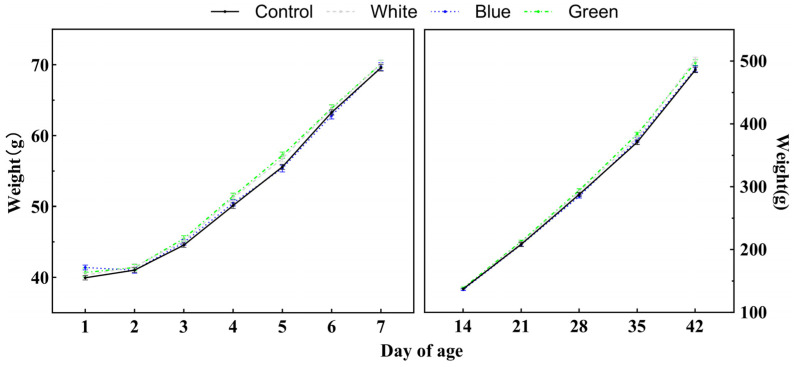
Body weight of chicks hatched under different incubation light treatments during the first 42 days.

There was no effect of batch on chick body weight, and the effect of age was significant (*p* < 0.05). The body weights of chicks in the control and light groups at different ages are shown in Figure 3. Body weight increased with age from D1 to D42. Meanwhile, the light treatments had no significant effect on body weight (*p* > 0.05), but further multiplied comparison (LSD test) showed that the average body weight on D5 and D42 were significantly different among four groups, as shown in Table 2. The average body weights of the green light group and white light group on D5 were significantly higher (*p* < 0.05) than that of the blue light group and the control group. Furthermore, the chicks of the white light group had significantly heavier body weight compared to the control and blue light groups (*p* < 0.05), and there was no difference between the green group and other groups (*p* > 0.05) on D42.

Body weight of sampling chicks had no significant difference between the four groups. Therefore, the absolute organ weight was compared. A significant effect of age on the weight of heart, liver, glandular stomach, gizzard, pancreas, spleen, thymus, and bursa of Fabricius (*p* < 0.05) was observed, and all showed a tendency to increase with age. Incubation light treatments only had a significant effect on the chick thymus weight (*p* < 0.05). Further multiplied comparison (LSD test) showed that chicks hatched under blue light incubation had a significantly lower thymus weight than that of the control group on D42 (*p* < 0.05), as shown in Table 2.

### 3.3. Blood Hormones of Chicks

There was no effect of incubation light treatments on IgA, IgY, IgM, and H/L ratio of post-hatch chicks. However, age had a significant impact on IgA, IgY, and H/L ratio (*p* < 0.05). The IgA and H/L ratio of chicks on D21 were significantly lower than those of chicks on D42. However, the IgY of chicks on D21 was significantly higher than that of chicks on D42. The plasma CORT was not affected by light treatments and age, while the interaction effect of light treatments and age did (*p* < 0.05). As shown in Table 3, the CORT concentration of chicks hatched in white light incubation and blue light incubation on D42 were significantly higher than that of control group (*p* < 0.05), and the green light was between.

### 3.4. Digestive Enzyme Activity of Chicks

There was no effect of age on the amylase and lipase activities of post-hatch chicks. However, incubation light treatments had a significant impact on amylase and lipase activities (*p* < 0.01). As shown in Table 3, the amylase and lipase activities of the control group, white light group, blue light group, and green light group gradually decreased on D21 and D42. The amylase activity of the green light group was significantly lower than that of the control group on D21 (*p* < 0.05). The lipase activity of the control group and all light groups were significantly different on D21 (*p* < 0.05). In addition, the amylase and lipase activities of control group and all light groups were significantly different on D42 (*p* < 0.05).

## 4. Discussion

The control of weight gain in laying hens is crucial for ensuring uniformity and managing the onset of sexual maturity. The ability to more closely manage chick growth provides producers with the ability to more closely manage the start of the production period. In the present study, chicks hatched in white light incubation had a growth advantage compared to chicks hatched in blue light and darkness incubation in the first 42 days, and the growth advantage of chicks hatched in green light incubation was slightly lower than that of the white light group but better than that of the blue light and control groups. It has been shown that providing both white and red light during incubation can improve layer quality across laying hens [24], which might in turn have affected the post-hatch weight gain of chicks. In a study on broilers, it was similarly reported that incubation under white light promoted weight gain in chicks [25]. In this study, chicks incubated under green light also exhibited better growth performance. Some researchers suggested that exposure to green and blue light during rearing may stimulate the secretion of testosterone and promote the growth of muscle fibres more effectively, leading to accelerated body growth [26]. Additionally, the authors summarized three possibilities for this, suggesting that green light stimuli increased the higher secretion of melatonin and somatotropic axis hormones, and enhanced the proliferation and differentiation of satellite cells in late embryonic stages. Higher plasma melatonin levels were indeed found in newly hatched chicks from green light incubation group in another study by our team [23]. However, compared to incubation in darkness, the effects of green light incubation on the post-hatch growth of RIR layer breeders were very limited, and the impact on weight diminished over time [27]. The weight changes in chicks incubated under green light in this study also exhibited similar characteristics.

It was reported that broilers hatched under other wavelength of light such as red during incubation significantly gained more weight after 18 days post-hatch than chicks hatched under blue light. It is due to more resting behaviour in the red-light group and more active behaviour in the blue-light group [28]. It seems that incubation light has a long-term impact on post-hatch growth performance through the change of specific behaviours. Therefore, a behaviour detector model was developed to assess the proximity of newly hatched chicks to feed and water resources in present study. It has been reported that the ability of chicks to find feed is influenced by the pecking sounds and movements of the mother hen [29]. The ability to find feed and water also reflects the environmental adaption of chicks. In commercial farms, however, chicks are simply transferred from hatchery and have to find feed and water by themselves. Previous studies have suggested that incubation light may influence the development of the left hemisphere of a chick’s brain, which is linked to behaviour control, including focused attention and positive emotions, including visual discrimination tasks, food-searching, vocal production, and recognition [8,30]. Incubation light also may lead to behavioural changes of post-hatch chicks, e.g., more frequent early feather pecking behaviour, representing social exploration [14] and greater feeding activity for the first 2 h after the lights turn on [31]. Chiandetti et al. [8] indicated that chicks incubated under the incandescent light bulb had a better orientation to feeders compared to chicks incubated in the dark. However, our results showed that the percentage of chicks accessing feed or water at different times on D1, D4, and D7 had no significant difference between different incubation light treatments. In the three days of first week, there was similar percentage of chicks accessing to feed and water in the morning, noon, and afternoon. It seems that chicks had better growth in prenatal white and green light groups, which may not be due to feeding behaviour.

During incubation, the embryo receives nutrients from the yolk and transitions to intestinal absorption after hatching, and the gastrointestinal tract induces growth during the early post-hatching period. The activity of digestive enzymes correlates with the efficiency of energy extraction from nutrients [32,33]. It was reported that the activities of jejunum amylase for broilers on D21 and D42 were 166.22 ± 7.51 U/g and 190.63 ± 11.30 U/g [34], respectively, and that for layers on 64 weeks was 170 U/g [35], which was similar to what we found in chicks. The activities of ileum lipase for broilers were reported to be 5.0 U/mg on D21 and 5.6 U/mg on D42 [36], and that for layers at 20 weeks was 6.15 U/mg [37], which was lower than that of the layer chicks in the present study. In a study investigating digestive enzyme activity in chickens suffering from stunting syndrome, it was found that the digestive enzyme activity in chickens with stunting syndrome was actually higher than that in chickens with normal body weight [38]. In the present study, the dark incubation chicks had higher activities of amylase and lipase than other light treatments incubation chicks on both D21 and D42, and this was also reflected in the growth performance of chicks in the control group.

It is reported that there is no difference in the relative weight of heart and liver for embryos between the green, blue, red, and white light treatments and dark during incubation [13,39]. Furthermore, as Li et al. [25] reported, providing blue, red, or white light illumination up to 12 h per day during incubation had no significant effect on the relative weight of spleen, liver and bursa of Fabricius, which was generally consistent with organ weight of chicks in this present study. However, thymus weight of chicks did appear to be affected by different incubation light treatments and was significantly lower in the blue incubation than in the control group on D42. Except the blue light group, the thymus weights of all other groups were similar to that reported by Liang et al. [40], which were 1.47 ± 0.22 g on D21 and 2.91 ± 0.22 g on D42. This indicated that monochromatic blue light during incubation inhibited the development of thymus for the post-hatch chicks, which may affect production and release of thymopoietin and thymosin, and consequently reduce disease and infection resistant. However, it was reported that monochromatic green or blue light during the rearing period promoted humoral immune function of chicks [41], and even more studies have reported that rearing under green or blue lights is positive for humoral and cellular immune responses [42,43]. However, the present study showed that light treatments during incubation had no impact on IgA, IgY, or IgM concentrations for post-hatch chicks. Likewise, other research showed no significant difference in IgY concentration of embryo incubated under dark, white and blue light treatments during incubation [25]. It seems that the effect of similar monochromatic light exposure on embryos and chicks was different, and photoperiod and intensity must also be considered.

Previous studies have shown that the application of the full-spectrum fluorescent light during the incubation period can reduce the environmental stress of the broilers after fledging, and the birds adapted to the environment more quickly and grew more rapidly [21,31]. The H/L ratio has been established as a reliable indicator of the amount of stress experienced by chickens, where lower values are indicative of lower stress levels [44]. The difference in H/L ratio of chicks hatched in different incubation lights was not observed in our study. The average H/L ratios of chicks were 0.40 ± 0.01 and 0.44 ± 0.01 on D21 and 42, respectively, which were slightly higher than that of the broiler hatched under white LED (0.279 ± 0.021) and dark (0.347 ± 0.021) on D14 [45]. Li et al. [25] also reported no differences in H/L ratio between incubation light treatments, and suggested that providing incubation light illumination up to 12 h per day did not induce stress levels during rearing period.

Corticosterone is the main glucocorticoid which is possible related to behavioural, physiological, and immune response changes in birds [46,47]. Archer and Mench [48] reported that the full-spectrum fluorescent light with 12L:12D photoperiod during incubation significantly reduced differences in corticosterone concentration about 0.06 ng/mL between before crating and 1 h after crating. There was an interaction effect of light treatments and age on plasma CORT of chicks on D42 in the present study, and the plasma CORT concentration of chicks incubated in darkness dropped from 5.25 ± 0.17 ng/mL on D21 to 3.61 ± 0.17 ng/mL on D42. However, the plasma CORT concentration of chicks was reported to increase from 2 ng/mL on D4 to approximately 5.2 ng/mL on D8, whereas in broilers, plasma CORT increased from 1.8 ng/mL on D4 to 2 ng/mL on D8 [49]. Plasma CORT of male layer chicks on D11 was reported as 7.2 ng/mL [50], and that of male layer chicks at 16 weeks of age was 4.2 ng/mL [51]. However, this might be related to the time of sampling within the day [52]. Plasma CORT concentration of chicks incubated under light was around 5 ng/mL both on D21 and D42, and light incubation maintained the plasma CORT levels of chicks stable in present study. Incubation light chicks may have a greater capability to acclimatize to the change of environments.

## 5. Conclusions

Incubation light did not affect general behaviour of chicks accessing to feed and water, but helped chicks maintain stable corticosterone levels and probably adapt to the change quickly. Utilizing white light during incubation appeared to have a positive effect on the early stage of chicks in terms of post-hatch growth and organ development. However, monochromatic blue light during the incubation period decreased the thymus weight of the chick, which may have negative effects. Therefore, further research is needed to clarify the underlying mechanism about prenatal different wavelength light, hormone release, post-hatch behaviour, and growth performance.

## Figures and Tables

**Table 1:** Performance of a chick accessing to the feeder or drinker recognition model.

Class	Precision/%	Recall/%	Average Precision/%	Mean Average Precision/%
Chick	94.33	88.18	95.92	97.64
Feeder	97.09	99.50	99.08	
Drinker	99.03	98.56	97.93	

**Table 2:** Body weight and organs weight of chicks hatched under different incubation light treatments.

Item	Age	Control	White	Blue	Green
Body Weight (g)	D1	39.96 ± 0.31	40.32 ± 0.37	41.41 ± 0.35	40.71 ± 0.62
	D5	55.59 ± 0.37 ^b^	56.78 ± 0.48 ^a^	55.38 ± 0.47 ^b^	57.25 ± 0.45 ^a^
	D42	486.04 ± 4.41 ^b^	502.45 ± 4.05 ^a^	487.59 ± 4.78 ^b^	497.70 ± 4.64 ^ab^
Spleen (g)	D21	0.34 ± 0.03	0.46 ± 0.06	0.33 ± 0.04	0.34 ± 0.05
	D42	1.08 ± 0.12	1.06 ± 0.05	1.07 ± 0.07	1.08 ± 0.05
Thymus (g)	D21	1.51 ± 0.02	1.44 ± 0.17	1.25 ± 0.09	1.44 ± 0.05
	D42	3.29 ± 0.31 ^a^	3.08 ± 0.20 ^ab^	2.57 ± 0.06 ^b^	3.00 ± 0.20 ^ab^
Bursa (g)	D21	1.11 ± 0.12	1.31 ± 0.10	1.24 ± 0.08	1.38 ± 0.11
	D42	2.74 ± 0.14	2.79 ± 0.19	2.84 ± 0.18	3.04 ± 0.27
Glandular stomach (g)	D21	1.54 ± 0.13	1.46 ± 0.05	1.37 ± 0.06	1.96 ± 0.37
	D42	2.57 ± 0.14	2.45 ± 0.27	2.54 ± 0.12	2.62 ± 0.14
Muscle stomach (g)	D21	6.23 ± 0.36	6.46 ± 0.59	7.86 ± 0.62	6.23 ± 0.31
	D42	12.31 ± 1.17	10.97 ± 1.08	11.67 ± 0.87	13.17 ± 0.57
Pancreas (g)	D21	0.86 ± 0.04	0.77 ± 0.10	0.74 ± 0.06	0.87 ± 0.04
	D42	1.36 ± 0.08	1.35 ± 0.09	1.50 ± 0.07	1.55 ± 0.08
Liver (g)	D21	6.52 ± 0.28	6.11 ± 0.24	5.54 ± 0.36	6.09 ± 0.44
	D42	12.49 ± 0.54	11.78 ± 0.36	11.89 ± 0.42	13.32 ± 0.77
Heart (g)	D21	1.58 ± 0.07	1.43 ± 0.08	1.32 ± 0.03	1.33 ± 0.22
	D42	2.45 ± 0.10	2.46 ± 0.09	2.38 ± 0.05	2.43 ± 0.11

^a, b^ Means in the same row with a different superscript differ significantly (*p* < 0.05).

**Table 3:** Blood hormones and digestive enzymes of chicks hatched under different incubation light treatments.

Item	Age	Control	White	Blue	Green
CORT (ng/mL)	D21	5.25 ± 0.17	5.06 ± 0.06	4.99 ± 0.23	4.79 ± 0.09
	D42	3.61 ± 0.17 ^b^	5.96 ± 0.69 ^a^	5.44 ± 0.71 ^a^	5.13 ± 0.20 ^ab^
H/L ratio	D21	0.40 ± 0.02	0.41 ± 0.01	0.39 ± 0.01	0.39 ± 0.02
	D42	0.41 ± 0.03	0.45 ± 0.02	0.44 ± 0.02	0.45 ± 0.03
IgA (g/L)	D21	2.278 ± 0.073	2.255 ± 0.045	2.13 ± 0.043	2.283 ± 0.061
	D42	3.360 ± 0.521	3.369 ± 0.521	3.234 ± 0.425	3.022 ± 0.355
IgY (g/L)	D21	4.201 ± 0.153	4.273 ± 0.116	4.300 ± 0.094	4.062 ± 0.073
	D42	3.117 ± 0.406	3.287 ± 0.462	3.357 ± 0.457	3.167 ± 0.430
IgM (g/L)	D21	1.697 ± 0.036	1.621 ± 0.042	1.602 ± 0.043	1.611 ± 0.044
	D42	1.627 ± 0.075	1.633 ± 0.060	1.599 ± 0.053	1.515 ± 0.028
Amylase in jejunum (U/g)	D21	159.60 ± 11.47 ^a^	138.75 ± 8.00 ^ab^	134.42 ± 7.14 ^ab^	106.44 ± 3.79 ^b^
	D42	200.47 ± 0.57 ^a^	146.68 ± 7.67 ^b^	113.16 ± 3.12 ^c^	94.06 ± 12.08 ^d^
Lipase in ileum (U/mg)	D21	15.90 ± 0.32 ^a^	13.57 ± 0.32 ^b^	11.77 ± 0.35 ^c^	8.26 ± 0.30 ^d^
	D42	13.32 ± 0.05 ^a^	11.94 ± 0.08 ^b^	11.64 ± 0.29 ^b^	11.37 ± 0.27 ^b^

^a–d^ Means in the same row with a different superscript differ significantly (*p* < 0.05).

## Data Availability

None of the data were deposited in an official repository. The data that support the study findings are available upon request.
